# Ultraviolet Photodetector Using Nanostructured Hexagonal Boron Nitride with Gold Nanoparticles

**DOI:** 10.3390/s25030759

**Published:** 2025-01-27

**Authors:** Dong Chan Kim, Hamin Park

**Affiliations:** 1 Department of Chemical, Biological, and Battery Engineering, Gachon University, Seongnam 13120, Republic of Korea; dckim@gachon.ac.kr; 2 Department of Semiconductor Engineering, Gachon University, Seongnam 13120, Republic of Korea; 3 Department of Electronic Engineering, Kwangwoon University, Seoul 01897, Republic of Korea

**Keywords:** hexagonal boron nitride, gold nanoparticle, ultraviolet, photodetector, photocurrent, plasmon resonance

## Abstract

Ultraviolet (UV) photodetectors play a crucial role in various applications, ranging from environmental monitoring to biomedical diagnostics. This paper presents the fabrication and characterization of a high-performance UV photodetector using hexagonal boron nitride (hBN) decorated with gold nanoparticles (AuNPs). The hBN flakes were mechanically exfoliated onto SiO_2_ substrates, and AuNPs were formed via thermal evaporation, resulting in the creation of a plasmonically active surface that enhanced light absorption and carrier dynamics. Raman spectroscopy, transmission electron microscopy, and electrical measurements were performed to comprehensively analyze the device structure and performance. The photodetector exhibited significantly improved photocurrent and responsivity under UV-B (306 nm) and UV-C (254 nm) illumination, with the responsivity reaching an increase of nearly two orders of magnitude compared to that of the pristine hBN device. These improvements are attributed to the synergistic effects of the wide bandgap of hBN and the localized surface plasmon resonance of the AuNPs. These findings demonstrate the potential of AuNP-decorated hBN for advanced UV photodetection applications and provide a pathway toward more efficient and miniaturized optoelectronic devices.

## 1. Introduction

Ultraviolet (UV) photodetectors have attracted significant attention owing to their critical roles in environmental monitoring, flame detection, communication systems, and biomedical sensing [1,2,3,4,5]. The ability to detect UV radiation is essential for various scientific, industrial, and societal reasons, such as monitoring ozone layer depletion, detecting high-energy radiation sources, and preventing UV-induced damage to materials and biological systems. Photodetectors that are sensitive to UV-B (306 nm) and UV-C (254 nm) wavelengths are crucial for detecting hazardous radiation. However, achieving high sensitivity, rapid response time, and stable operation using UV photodetectors is challenging because of the inherent limitations of the material properties and device architectures.

Conventionally, wide-bandgap semiconductors such as GaN, ZnO, SiC, and GaO are the main materials for UV detection [6,7,8,9]. However, despite their effectiveness, their rigid and bulky characteristics pose challenges to their integration into flexible and scalable device architectures. According to Moore’s Law, electronic devices have become highly integrated, with dimensions reaching just a few nanometers or even sub-nanometer scales [10]. Consequently, photodetectors must also be miniaturized to the nanoscale to achieve a high performance and seamless integration within advanced technological systems. Since the discovery of graphene in 2004, research on two-dimensional (2D) materials has surged, leading to extensive exploration of various 2D-based materials. Over the past decade, the unique properties of single-layer graphene have garnered significant attention, positioning 2D materials at the forefront of research due to their potential applications in emerging nanoscale optoelectronic and electronic devices. Numerous studies have been conducted to investigate the physical characteristics and applications of these materials, focusing on phenomena such as energy band structures, carrier transport properties, the quantum Hall effect, and the spin–orbit coupling [11,12,13,14]. Hexagonal boron nitride (hBN) is a layered crystal with a hexagonal structure similar to that of graphite [15,16]. In its lattice, boron and nitrogen atoms are alternately arranged and bonded in-plane via sp^2^ hybridization, forming a hexagonal network. Multiple layers of this lattice stack together to create bulk hBN. The sp^2^ bonding of BN results in robust in-plane σ bonds, which contribute to its high thermal conductivity and chemical stability. Meanwhile, the unoccupied π orbitals render hBN electrically insulating with a wide bandgap of approximately 6 eV. Therefore, hBN has attracted considerable interest for UV photodetection applications [17,18,19,20,21]. Its 2D property, high transparency in the visible range, exceptional thermal stability, and chemical inertness make it an ideal candidate for high-performance UV photodetectors.

Despite their satisfactory properties, pristine hBN-based devices often exhibit low responsivity and limited photocurrent generation. This performance limitation is primarily attributed to the weak light absorption in the UV range and the inefficient charge carrier transport within the material. Recent studies have focused on overcoming these limitations through surface engineering, structural modifications, and incorporation of plasmonic nanostructures [22,23,24]. These approaches aim to enhance optical and electronic interactions within the photodetector, improving the device performance. Among various enhancement methods, decoration with nanoparticles has emerged as a particularly effective approach [25,26]. Localized surface plasmon resonance (LSPR) is a phenomenon observed in metallic nanoparticles or spatially confined islands, where conduction electrons collectively oscillate in resonance with incident electromagnetic waves. This resonance condition is highly sensitive to the surrounding medium’s dielectric constant and refractive index [27,28,29]. LSPR generates enhanced electromagnetic fields near the nanoparticle surface, significantly boosting light absorption and scattering. The confined electric field amplifies interactions with nearby materials, making it particularly advantageous for sensitive detection applications. In nanostructures, these localized fields contribute to higher photocarrier generation rates and improved charge transport dynamics. The interplay between electron oscillation and light absorption under LSPR facilitates enhanced optical properties, such as increased responsivity in photodetectors. The nanoscale tuning of these systems can optimize resonance conditions, providing a versatile platform for advanced optoelectronic applications. This plasmonic effect significantly facilitates light absorption and photocarrier generation in the underlying hBN layer, thereby improving the overall performance of the UV photodetectors. Furthermore, nanoparticles serve as efficient charge trapping and transfer centers, facilitating the separation and transport of photo-generated carriers and improving the response of the device to UV radiation.

In this study, we fabricate and characterize a UV photodetector using hBN decorated with gold nanoparticles (AuNPs), deposited under high vacuum conditions without the use of any solvent. The device structure is designed to leverage the synergistic effects of the wide-bandgap properties of hBN and the plasmonic enhancements of AuNPs. The photocurrent and responsivity of the device under different UV illumination conditions, including UV-B and UV-C wavelengths, are investigated. A comparative analysis of pristine hBN and AuNP-decorated hBN devices highlights the significant improvement in the photodetection performance achieved using this plasmonic decoration approach.

## 2. Materials and Methods

### 2.1. Materials

The fabrication of the UV photodetector device began with the mechanical exfoliation of the hBN flake (33 nm) from bulk hBN crystals supplied by 2D Semiconductors Corporation. Exfoliation was performed on a 90 nm silicon dioxide (SiO_2_) substrate. Prior to the exfoliation, the SiO_2_ substrate was cleaned to ensure optimal surface conditions. The cleaning process involved immersion in a piranha solution (H_2_SO_4_:H_2_O_2_ = 2:1) to remove organic contaminants, followed by an oxygen plasma treatment to enhance surface hydrophilicity. After exfoliation, the hBN flakes were annealed at 400 °C in a diluted H_2_ atmosphere to eliminate residual scotch tape adhesive, thereby ensuring a pristine surface quality. Titanium (Ti) electrodes with a 100 nm thickness were thermally evaporated and patterned with channel dimensions of 12 µm in width and 5 µm in length. The patterning process was carried out using a negative photoresist (NR9, Futurrex, Franklin, NJ, USA) in conjunction with a mask aligner (MA6, Karl Suss, Garching, Germany). Raman spectra were recorded using a high-resolution dispersive Raman microscope (514 nm Ar ion laser, ARAMIS, Horiba Jobin Yvon, Kyoto, Japan) with a laser spot size of 2 µm to ensure accurate measurements of the flakes. Transmission electron microscopy (TEM) images were captured using a Cs-corrected Titan TEM instrument (FEI) to provide high-resolution insights into the atomic structure of the hBN layers. AuNPs were formed on the hBN surface using a thermal evaporator (KVE-T2010, KVT, Gimpo, Republic of Korea). A thin gold film with a thickness of 3 nm was deposited at a rate of 0.1 nm/s under high vacuum conditions (~5 × 10^−6^ Torr). Due to the agglomeration of gold at atomically thin thicknesses, this process led to the formation of AuNPs. This thermal process was employed to produce high-quality AuNPs in a clean environment without the use of any solvents [30,31]. The formation of AuNPs was analyzed using scanning electron microscopy (SEM) (Magellan 400, FEI, Hillsboro, OR, USA) after coating the surface with a 5 nm thick layer of osmium (Os) to mitigate electron charging.

### 2.2. Methods

The performance of the photodetector under UV illumination was evaluated using a UV lamp (Dongseo Science, Dangjin, Republic of Korea) that emitted at two specific wavelengths: UV-B (306 nm) with an intensity of 1107 µW/cm^2^ and UV-C (254 nm) with an intensity of 1422 µW/cm^2^. Electrical measurements were performed using a chamber probe station (MST-1000B, MS TECH, Hwaseong, Republic of Korea) integrated with a parameter analyzer (4200 SCS, Tektronix, Beaverton, OR, USA) to ensure precise control and data acquisition during the experiments. During the evaluation of the optoelectronic performance, the UV lamp was fixed 5 cm directly above the device placed on the probe station stage.

## 3. Results and Discussion

**Figure 1:**
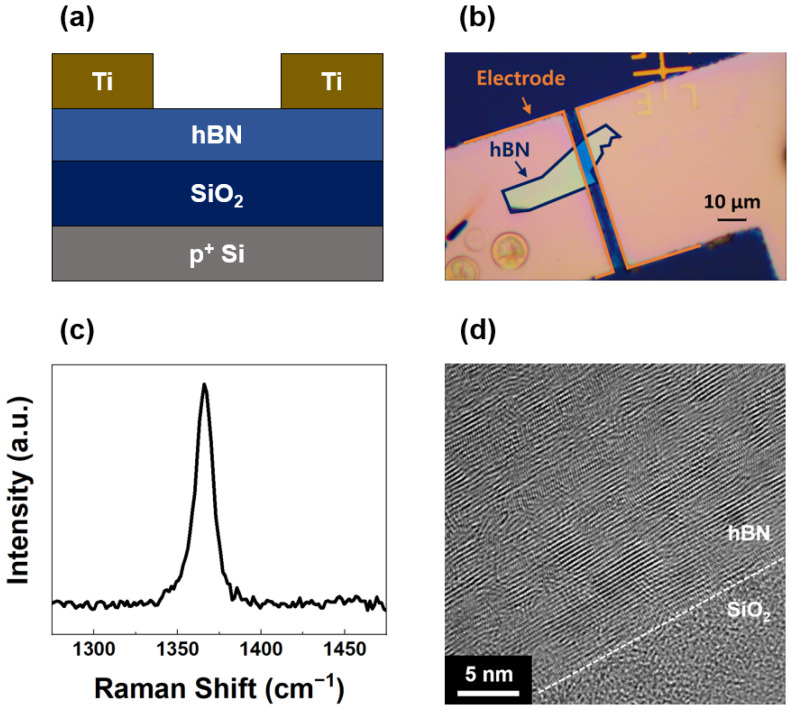
(**a**) Cross-sectional schematic and (**b**) optical microscopic image of the hBN photodetector. (**c**) Raman spectrum of the hBN flake. (**d**) Cross-sectional TEM image of the hBN-SiO_2_ interface.

Figure 1a,b show a cross-sectional schematic and an optical microscopic image of the photodetector device, respectively, which consists of the hBN flake and the top electrodes on a SiO_2_/Si substrate. The blue line outlines the shape of the hBN flake, while the orange line represents the shape of the electrodes. Figure 1c shows the Raman spectrum of the hBN flake, exhibiting the peak at 1366 cm^−1^, corresponding to the in-plane E_2g_ phonon mode. In contrast to graphene, which exhibits G, D, and 2D Raman bands, hBN exhibits only a G band corresponding to the E_2g_ vibrational mode. The absence of a D band in hBN is attributed to the lack of a Kohn anomaly [32]. The Raman peak observed at 1366 cm^−1^ confirms that the hBN layer consists of multiple layers, more than four. Additionally, the sharp peak without additional peaks demonstrated the crystalline quality with minimal contamination from the exfoliation and annealing processes. Figure 1d shows a cross-sectional TEM image of the hBN-SiO_2_ interface. The interlayer van der Waals distances of hBN observed in the image are 0.33 nm along the c-axis of the crystal. The layered van der Waals structure of hBN and the well-defined interface between hBN and SiO_2_ are clearly visible. The results presented in Figure 1a–d collectively show the high quality of the hBN flake and its compatibility with the architecture of the device. The exfoliation and annealing processes successfully produced defect-free, uniform flakes with strong adhesion to the substrate. The Raman and TEM analyses validated the crystalline property and structural integrity of hBN which are critical for achieving high performance in UV photodetection.

**Figure 2:**
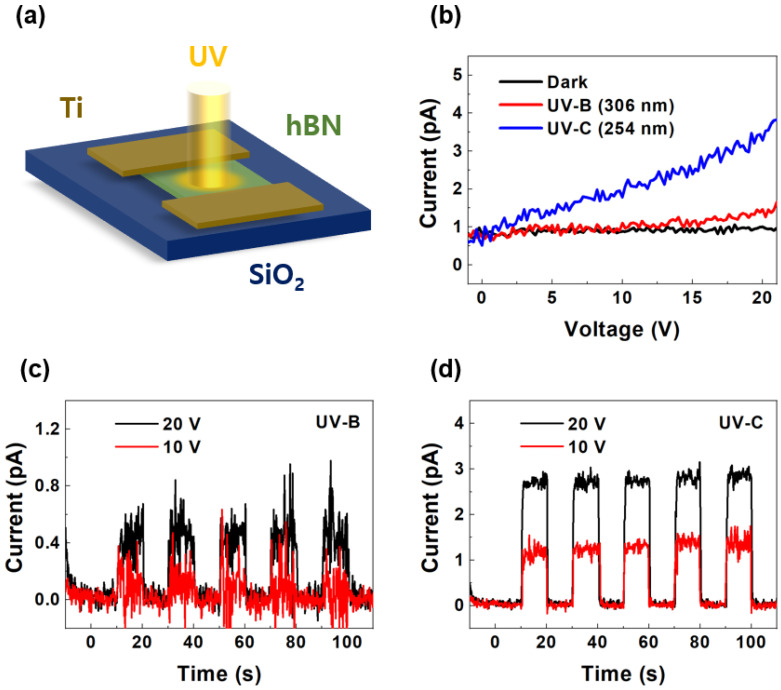
(**a**) Schematic of the device and measurement configuration of the pristine hBN device under UV illumination. (**b**) Current–voltage characteristics measured under dark conditions (black), UV-B illumination (red), and UV-C illumination (blue). Time-dependent photocurrent responses under (**c**) UV-B and (**d**) UV-C illumination at bias voltages of 10 V (red) and 20 V (black).

Figure 2 shows the performance of the pristine hBN-based UV photodetector under UV-B (306 nm) and UV-C (254 nm) illumination. Figure 2a depicts the device and measurement configuration, in which UV light irradiates the hBN surface with an applied voltage through the Ti electrodes. Figure 2b shows the current–voltage (I-V) characteristics of the device under dark conditions (black), UV-B illumination (red), and UV-C illumination (blue). The photocurrent increased more under UV illumination than under dark conditions, with a higher response observed for UV-C because of its higher photon energy. The results confirm the sensitivity of the device to UV wavelengths and the effective charge carrier generation under UV exposure. Furthermore, the current and voltage exhibited a linear relationship, confirming the drift of photocarriers under the applied electric field. Figure 2c,d show the time-dependent photocurrent responses of the device under UV-B and UV-C illumination, respectively, at bias voltages of 10 V (red) and 20 V (black). The UV lamp was turned on and off at 10 s intervals, and the current flowing through the UV photodetector was continuously measured during this process. The bandgap of hBN was ~6 eV, whereas the UV-B and UV-C energy values were 4.1 and 4.9 eV, respectively. These energy values are insufficient to excite electrons from the valence band to the conduction band in hBN, as they do not overcome the bandgap of hBN. Consequently, direct band-to-band photo-excitation does not occur under UB-B and UV-C illumination, resulting in a low photocurrent and responsivity. Nevertheless, all cases demonstrated consistent and repeatable photocurrent responses, with higher photocurrent values observed at 20 V than at 10 V. The measured current exhibited a flickering noise, which was noticeable because the current level was extremely low in the pA range. The periodic on–off behavior in both cases indicates the reversibility and stability of the device during repeated UV exposure cycles. However, the photodetector with the pristine hBN flake exhibited low photocurrent levels and responsivities.

**Figure 3:**
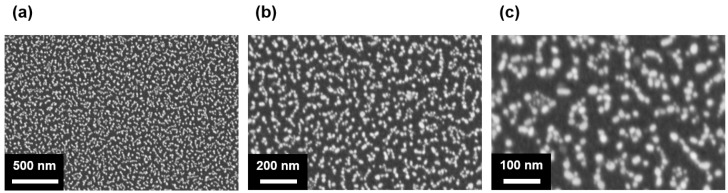
SEM images of AuNPs on the hBN surface with scale bars of (**a**) 500 nm, (**b**) 200 nm, and (**c**) 100 nm.

Noble metal nanoparticles exhibit significantly enhanced optical properties due to LSPR. When exposed to light, the collective oscillation of the electrons generates a highly concentrated electromagnetic field within nanoscale gaps of the metallic nanostructure. Achieving a strong electromagnetic field near noble metals requires nanoscale separation, as the field intensity is inversely proportional to the size of the metallic gap [33]. Therefore, we deposited AuNPs on the hBN surface via thermal evaporation of gold to improve the photocurrent and responsivity based on LSPR. Figure 3a–c are SEM images of the AuNPs formed on the SiO_2_ substrate at different resolutions. These images were obtained after depositing a 3 nm thick gold film, which subsequently agglomerated to form nanoparticles. The AuNPs were uniformly dispersed, having a nanoscale size (~15 nm) and distribution with a number density of 8.5 × 10^10^ cm^−2^ across the substrate. This uniformity is crucial to ensure consistent plasmonic enhancements throughout the device. Furthermore, the SEM images confirmed the spherical shape of the AuNPs and highlighted their nanoscale dimensions. These characteristics are essential for achieving the desired LSPR properties that amplify the UV light absorption and improve the charge carrier dynamics.

**Figure 4:**
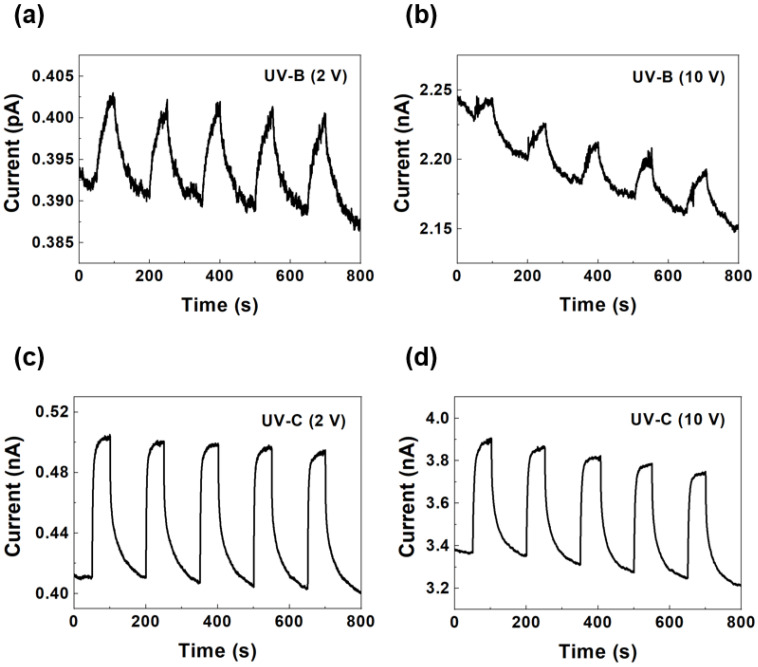
Time-dependent photocurrent responses of AuNP-decorated device under UV-B illumination at bias voltages of (**a**) 2 V and (**b**) 10 V, and under UV-C illumination at bias voltages of (**c**) 2 V and (**d**) 10 V.

Figure 4 shows the photocurrent of the AuNP-decorated hBN photodetector under repeated exposure to UV-B and UV-C illumination at different bias voltages. The UV lamp cycled on and off every 10 s, while the current through the UV photodetector was continuously monitored throughout the process. Figure 4a,b show the time-dependent photocurrent responses under UV-B illumination at bias voltages of 2 V and 10 V, respectively. The device exhibited stable and repeatable photocurrent behavior over multiple on–off cycles. The higher photocurrent observed at 10 V rather than at 2 V reflects the enhanced charge carrier separation and transport efficiency at a higher electric field across the hBN channel. Figure 4c,d show the time-dependent photocurrent responses under UV-C illumination at bias voltages of 2 V and 10 V, respectively. Similarly to the UV-B response, the device exhibited consistent on–off switching behavior with higher photocurrent values under UV-C illumination, attributable to the higher photon energy from the shorter wavelength of UV-C light. The well-defined transitions between the on and off states highlight the rapid response and recovery times of the photodetectors. For the 10 V bias cases, the current decreased over the cycles owing to the Joule heating effect. Higher temperatures induce lower carrier mobilities in 2D materials, such as graphene, MoS_2_, MoSe_2_, WS_2_, and WSe_2_ [34,35,36,37,38]. Therefore, the mobility of the UV-generated carriers decreased as the number of on–off cycles increased at a high bias voltage.

**Figure 5:**
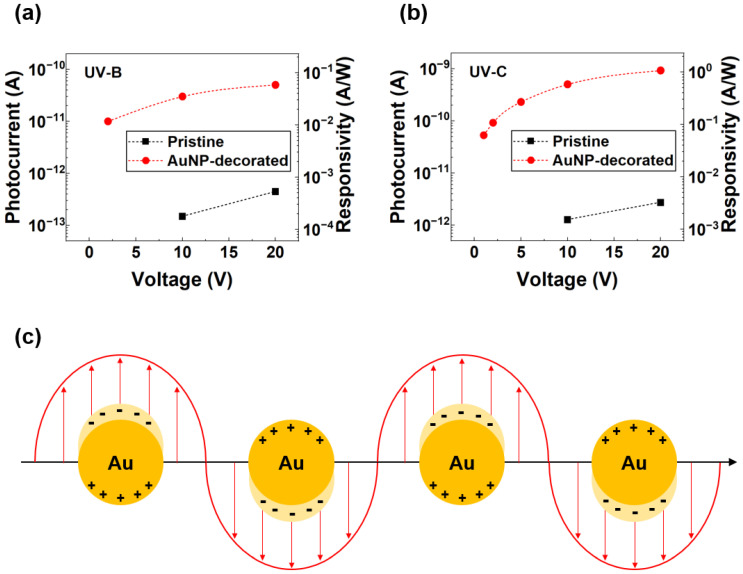
Photocurrent and responsivity under (**a**) UV-B and (**b**) UV-C radiation as functions of the applied voltage for pristine devices (black squares) and AuNP-decorated devices (red circles). (**c**) Schematic of localized surface plasmon resonance (LSPR) between AuNPs under UV illumination.

Figure 5a,b show the photocurrent and responsivity as functions of the applied voltage for the pristine (black squares) and AuNP-decorated (red circles) devices under UV-B and UV-C radiation, respectively. The responsivity values were calculated as *R* = *I_P_*/*P*, where *R*, *I_P_*, and *P* are the responsivity, photocurrent, and power, respectively. The AuNP-decorated device exhibited a significantly higher photocurrent and responsivity across the voltage range, underscoring the plasmonic enhancement effect introduced by the AuNPs. In particular, the responsivity reached an increase of nearly two orders of magnitude compared to that of the pristine device at a bias of 10 V. Specifically, under UV-B illumination, the responsivity improved by 2.0 × 10^2^ times, and, under UV-C illumination, it improved by 3.9 × 10^2^ times at the same bias. The plasmonic enhancement by AuNPs significantly improves the ability of the device to detect UV light, making it a promising candidate for practical applications of high-performance UV photodetection. The stability further reinforces the potential of the device for long-term practical use. Figure 5b shows the LSPR between AuNPs on the hBN surface under UV illumination. LSPR promotes optical absorption, which increases the generation of charge carriers that contribute to the photocurrent. Furthermore, LSPR is directly influenced by the amplification of the local electric field (red arrows in Figure 5c), which promotes the separation of the generated electron-hole pairs, thereby further improving the photocurrent [26,27].

## 4. Conclusions

In this study, we fabricated and characterized a high-performance UV photodetector using AuNP-decorated hBN. The results highlight the significant enhancements in photocurrent and responsivity achieved through the integration of AuNPs, which are attributed to their LSPR effects. The hybrid hBN-AuNP device exhibited stable, repeatable, and sensitive operations under UV-B and UV-C illumination, making it a promising candidate for advanced UV detection applications. Comprehensive analyses, including structural characterization via Raman spectroscopy, TEM, and SEM, confirmed the high quality of the materials and their effective integration. Electrical measurements further demonstrated the robustness and reliability of the device under prolonged UV exposure, demonstrating its potential for practical applications. A key achievement of this study is the nearly two orders of magnitude improvement in responsivity compared to pristine hBN devices, making the first demonstration of an hBN photodetector enhanced with AuNPs. This enhancement highlights the effectiveness of plasmonic nanostructures in overcoming the inherent limitations of hBN, such as weak light absorption and inefficient charge carrier transport. The integration of hBN with the plasmonic properties of AuNPs provides a versatile platform for the development of advanced photodetectors. Future work should include other plasmonic materials and 2D substrates to open new pathways for designing next-generation UV photodetectors with improved efficiency and versatility.

## Data Availability

The data presented in this study are available upon request from the corresponding author.
